# Cyclohexylamine, the principal metabolite of cyclamate, contracts the epididymal vas deferens of rats by affecting endogenous catecholamine release via postsynaptic α_1A_- and presynaptic α_2_-adrenoceptors in a calcium-dependent manner

**DOI:** 10.37796/2211-8039.1653

**Published:** 2025-06-01

**Authors:** Yuh-Fung Chen, Yu-Wen Wang, Huei-Yann Tsai

**Affiliations:** a Department of Pharmacology, China Medical University, Taichung 404328, Taiwan, ROC; b Department of Pharmacy, China Medical University Beigang Hospital, Yunlin County 651012, Taiwan, ROC; c Department of Pharmacy, China Medical University Hospital, Taichung 404327, Taiwan, ROC

**Keywords:** α_1A_-Adrenergic receptors, α_2_-Adrenergic, Cyclohexylamine, Isolated rat epididymal vas deferens, Calcium-dependent

## Abstract

**Background:**

Cyclohexylamine (CHA) is a principal metabolite of cyclamate, which was once one of the most prominently consumed non-sugar sweeteners. Earlier studies suggested that long-term use of cyclamate might be carcinogenic and genotoxic; however, no consistent evidence supports an association between cyclamate and cancer risk. However, this issue remains interesting. Cyclamate can be metabolized to CHA by the intestinal bacteria in humans and some animals. Previous reports indicated CHA could induce atrophy of rat testes and affect rat fertility, as well as contract rat vas deferens. However, the contractile mechanisms of CHA on rat vas deferens remains poorly understood. This study investigated the contractile mechanisms of CHA on the isolated rat epididymal portion of the vas deferens.

**Methods:**

Male S.D. rats weighing between 200 g and 250 g were used. The isolated epididymal portion of rat vas deferens was added to calcium-channel blockers, calcium-free conditions or various concentrations (1 × 10^−8^ M−1 × 10^−5^ M) of adrenergic antagonists and CHA (1 × 10^−4^ M).

**Results:**

CHA (1 × 10^−5^ M−1 × 10^−1^ M) evoked a concentration-dependent contraction. Calcium-channel blocker, nifedipine (1 × 10^−8^M−1 × 10^−6^ M) or verapamil (1 × 10^−8^M−1 × 10^−5^ M) pretreatment, dose-dependently attenuated the CHA (1 × 10^−4^ M)-induced contraction. The calcium-free condition completely blocked CHA (1 × 10^−4^ M)-induced contraction. Prazosin (1 × 10^−8^M−1 × 10^−6^ M) or yohimbine (1 × 10^−7^ M−1 × 10^−5^ M) pretreatment or treatment could inhibit contractions evoked by CHA (1 × 10^−4^ M) in a dose-dependent manner. Moreover, the effect of CHA (1 × 10^−4^ M) was entirely blocked by combining prazosin and yohimbine pretreatment. WB4101 (1 × 10^−8^ M) could completely inhibit the contractions induced by CHA (1 × 10^−4^ M). CEC (1 × 10^−8^ M−1 × 10^−4^ M) showed no significant inhibitory effect on the contractile tension but reduced the frequency induced by CHA (1 × 10^−4^ M). Moreover, reserpine (1 × 10^−5^ M) showed a significant inhibition on the contractions of CHA (1 × 10^−4^ M).

**Conclusions:**

From the above results, CHA-contracts epididymal vas deferens of rats by affecting endogenous catecholamine release via postsynaptic α_1A_- and presynaptic α_2_- adrenoceptors and are calcium-dependent.

## 1. Introduction

Cyclohexylamine (CHA) is the principal metabolite of cyclamate, a non-sugar sweetener (NSS). Cyclamate was first introduced to the marketplace in 1951 [1] and was used as a low-calorie sweetener in the United States [2]. Cyclamate is 30 times sweeter than sucrose and has become a sugar alternative to sucrose [3]. However, cyclamates were banned by the FDA as a food ingredient in 1969 because the saccharin/cyclamate mixture was shown to cause cancer in laboratory rats, and the primary concern was that it could be toxic to some individuals who appear to metabolize cyclamate to cyclohexylamine [2]. Nevertheless, many countries did not follow the decision of the U.S. to ban cyclamate, which continued to be used as a food additive [4]. Although cyclamate has been comprehensively reviewed multiple times by agencies worldwide for genotoxicity and carcinogenicity, the consistent conclusion was that there is no evidence of genotoxicity and carcinogenicity by cyclamate [4]. However, the safety of cyclamate is still controversial and continues to be of interest.

Previous reports [5–8] indicate that CHA is the primary metabolite of the sweetener cyclamate. It has been shown [9] that bacteria in the intestines of humans, rabbits, and rats can convert cyclamate to CHA. Therefore, many scholars have emphasized research on the pharmacological toxicity of CHA. According to several studies [10,11], CHA affects blood pressure; at low doses, CHA increases blood pressure, which may be related to the effect of norepinephrine release and acts on the α_1_-adrenoceptor, whereas at high doses or with prolonged use of CHA, a decrease in blood pressure was observed, which may be a result of the emptying of norepinephrine. Furthermore, CHA was cytotoxic, causing testicular atrophy in vivo and cell culture experiments in rats [6,12–14]. Histological changes revealed that more than 75 % of the seminiferous tubules were damaged and that most germ cells were emptied and depleted [13]. In addition, histopathological studies revealed a focal basal vacuolar state in the Sertoli cell cytoplasm [12].

It was reported that CHA caused the rat vas deferens contractile response, mediated by activating α-adrenergic and cholinergic receptors [15]. Due to the presence of noradrenergic receptors in the rat vas deferens and the contractile mechanism of CHA on rat vas deferens remaining poorly understood, the present experiment is expected to investigate the CHA-induced contraction further.

## 2. Materials and methods

### 2.1. Materials

Cyclohexylamine (CHA), EGTA, DMSO, prazosin, nifedipine, verapamil, yohimbine, WB4101, chloroethyl clonidine (CEC), and reserpine were purchased from Sigma Aldrich Research Biochemicals, Inc. (“Sigma-RBI), USA. Zoletil^®^ was purchased from Virbac Laboratories (Carros, France). Reserpine and nifedipine were dissolved in Krebs-Ringer solution in the presence of DMSO. Other drugs were dissolved in Krebs-Ringer solution. The composition of Krebs-Ringer solution is expressed as follows (mM): NaCl 119; NaHCO_3_ 24.9; d-Glucose 11; KH_2_PO_4_ 1.2; KCl 4.6; MgSO_4_·7H_2_O 1.2; CaCl_2_·2H_2_O 1.5. The composition of Ca^2+^-free Krebs-Ringer solution was the same as Krebs-Ringer solution except 1 mM/L EGTA replaced CaCl_2_·H_2_O.

### 2.2. Ethics statement and preparation of the isolated epididymal vas deferens

Male Sprague Dawley (SD) rats weighing between 200 and 250 g were purchased from the National Laboratory Animal Center (NLAC), Taipei. Animals were fed with standard chow and housed in standard cages with a 12 h inverted light–dark cycle. The experimental protocol was approved by the Institutional Animal Care and Use Committee (IACUC), China Medical University (permit number: 2019-332). Male SD rats were anesthetized with Zoletil® (50 mg/kg, i.p.) and sacrificed by cervical dislocation. The vas deferens were removed and cleaned of the surrounding connective tissue and blood vessels. Only the epididymal portion (0.6–1 cm) of vas deferens was used. The tissues were mounted into 5 ml Magnusorgan baths containing Krebs-Ringer solution at 37 °C and bubbled with 95 % O_2_/5 % CO_2_. In addition, the vas deferens were connected to a Grass Isometric displacement transducer FT 03 linking Gould transducer amplifier, and the isometric contractions of vas deferens were recorded by Gould 2600s. Preparations were loaded with 1.5 g resting tensions and then refreshed the Krebs-Ringer solution every 10 min. Preparations were equilibrated for 1 h.

### 2.3. Effects of cyclohexylamine (CHA) on the isolated rat epididymal vas deferens

A single dose of CHA (1 × 10^−5^ M−1 × 10^−1^ M) was added to the organ bath for 20 min respectively and then washed out until the response returned to baseline. The response was observed. Calcium antagonists, nifedipine (1 × 10^−6^ M−1 × 10^−4^ M) or verapamil (1 × 10^−8^ M−1 × 10^−6^ M), were pretreated for 20 min before CHA (1 × 10^−4^ M) was added. Moreover, Krebs-Ringer solution was replaced by Ca^2+^-free Krebs-Ringer solution for 20 min, and then CHA was added. The effects of calcium antagonists and Ca^2+^-free conditions on the CHA-induced contraction in the isolated rat epididymal vas deferens were observed.

### 2.4. Effects of α-adrenergic antagonist: prazosin or yohimbine or combination of prazosin and yohimbine on CHA-induced isolated rat epididymal vas deferens

Different concentrations of prazosin (1 × 10^−8^ M−1 × 10^−6^ M) or yohimbine (1 × 10^−7^ M−1 × 10^−5^ M) were pretreated for 10 min before CHA (1 × 10^−4^ M) was administered. Additionally, while contractions were evoked by CHA (1 × 10^−4^ M), different concentrations of prazosin (1 × 10^−6^ M-1 × 10^−4^ M) or yohimbine (1 × 10^−7^ M−1 × 10^−5^ M) were treated in the sixth minute. The effects of α_1_- and α_2_-antagonists on the contractions evoked by CHA in isolated rat epididymal vas deferens were observed.

The combination of prazosin (1 × 10^−8^ M) and yohimbine (1 × 10^−6^ M) was pretreated for 10 min before adding CHA (1 × 10^−4^ M). The effect of the combination of prazosin and yohimbine on the contractions evoked by CHA (1 × 10^−4^ M) was observed and recorded.

Different concentrations of the α_1A_-adrenergic antagonist WB4101 (1 × 10^−9^ M, 3 × 10^−9^ M, 1 × 10^−8^ M), the α_1B_-adrenergic antagonist, chloroethylclonidine (CEC) (1 × 10^−8^ M−1 × 10^−4^ M) were pretreated for 10 min, and the catecholamine depletor, reserpine (1 × 10^−6^ M−1 × 10^−4^ M) were pretreated for 2 h before CHA (1 × 10^−4^ M) was added.

### 2.5. Statistical analysis

The results were expressed as mean ± S.E. The differences between mean values were compared using one-way ANOVA (post hoc test with Duncan’s test) or the Student *t*-test. When *P* < 0.05, it was considered statistically significant.

## 3. Results

### 3.1. Effects of different concentrations of CHA on the isolated rat proximal epididymal vas deferens

**Fig. 1:**
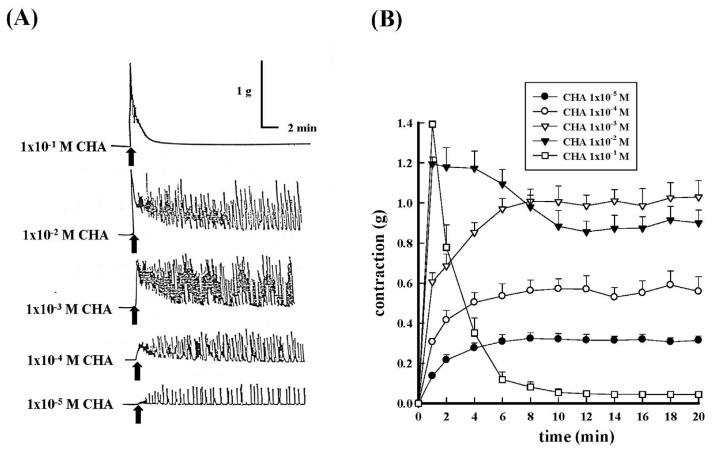
Effects of different concentrations of cyclohexylamine (CHA)-induced contraction of the isolated rat epididymal vas deferens. 1A represented the contraction trace and 1B represented the contraction force (g) change of different concentration (1 × 10^−5^ M~1 × 10^−1^ M) of CHA treatment of the isolated rat epididymal vas deferens.

Different concentrations (1 × 10^−5^ M−1 × 10^−1^ M) of CHA dose-dependently contracted the isolated rat proximal epididymal vas deferens as shown in Fig. 1A. The maximal contraction tension was 0.32 ± 0.03 g; 0.57 ± 0.05 g; 0.99 ± 0.05 g; 1.20 ± 0.03 g; 1.39 ± 0.08 g, respectively (Fig. 1B). The maximum contraction could be reached when the concentration of CHA was 1 × 10^−3^ M, and the strong contraction was caused when the CHA concentration was more than 1 × 10^−2^ M. After 8 min of the reaction time, the contraction reaction produced by CHA began to expand. When the concentration of CHA was 1 × 10^−1^ M, there was a rapid and intense phasic phase at the beginning, which could be up to about 1.4 g (Fig. 1B). After that, there was a significant relaxation immediately, and there was no longer any tonic contraction.

### 3.2. Effects of calcium antagonists and calcium-free on the CHA-induced contraction of the isolated rat proximal epididymal rat vas deferens

**Fig. 2:**
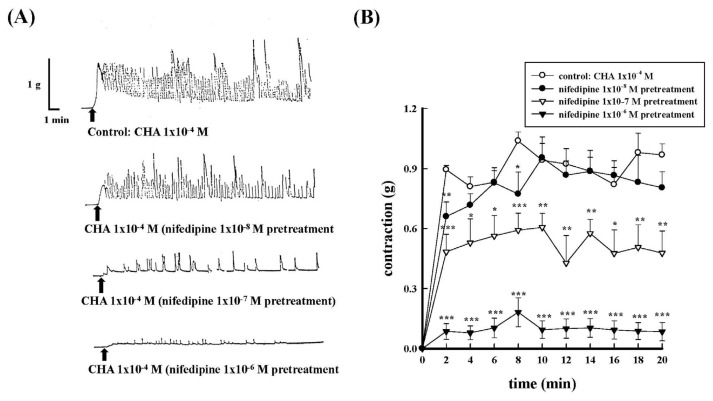
Effects of different concentrations of nifedipine pretreatment on CHA-induced contraction of the isolated rat epididymal vas deferens. 2A represented the contraction trace of isolated rat epididymal vas deferens and the contraction force (g) change (2B) of nifedipine (1 × 10^−8^ M ~1 × 10^−6^ M) pretreatment on CHA (1 × 10^−4^ M)-induced contraction. Nifedipine (1 × 10^−6^ M) pretreatment significantly inhibited the CHA-induced contraction.

**Fig. 3:**
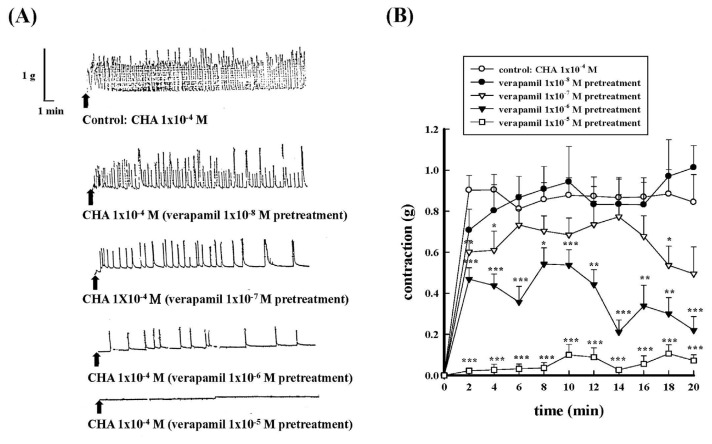
Effects of different concentrations of verapamil pretreatment on CHA-induced contraction of the isolated rat epididymal vas deferens. 3A represented the contraction trace of isolated rat epididymal vas deferens and the contraction force (g) change of verapamil (1 × 10^−8^M~1 × 10^−5^ M) pretreatment on CHA (1 × 10^−4^ M)-induced contraction (3B). Verapamil (1 × 10^−5^ M) pretreatment greatly inhibited the CHA-induced contraction.

In order to evaluate the role of calcium on the contractile effects of CHA on isolated rat proximal epididymal vas deferens, different calcium channel blockers and calcium-free conditions were used. Different concentrations of nifedipine (1 × 10^−8^ M−1 × 10^−6^ M) or verapamil (1 × 10^−8^ M−1 × 10^−5^ M) pretreatment dose-dependently inhibited CHA (1 × 10^−4^ M)-induced contraction of the isolated rat proximal epididymal rat vas deferens as shown in Figs. 2 and 3. Figs. 2A and 3A represented the contraction trace, and Figs. 2B and 3B represented nifedipine’s contraction force change (g) and verapamil pretreatment on CHA-induced contraction, respectively.

**Fig. 4:**
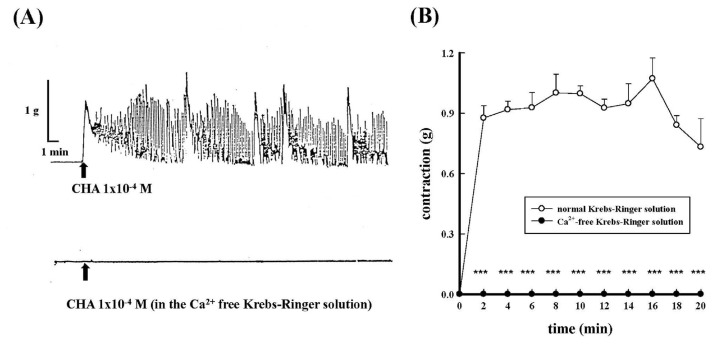
Effects of calcium-free condition on CHA-induced contraction of the isolated rat epididymal vas deferens. 4A represented the contraction trace of isolated rat epididymal vas deferens and the contraction force (g) change of calcium-free condition on CHA (1 × 10^−4^ M)-induced contraction (4B). Calcium-free condition entirely inhibited the CHA-induced contraction.

After replacing the Krebs-Ringer solution with a Ca^2+^-free Krebs-Ringer solution containing EGTA (1 mM/L), it was found that CHA (1 × 10^−4^ M) did not produce a contractile response in the epididymal rat vas deferens as shown in Fig. 4. ***P < 0.001 compared with the CHA group.

### 3.3. Effect of α_1_-adrenergic antagonist prazosin and α_2_-adrenergic antagonist yohimbine on the CHA-induced contraction of the isolated rat proximal epididymal rat vas deferens

Because α-adrenergic receptors mediate the contraction of the rat proximal epididymal vas deferens, the α_1_-adrenergic antagonist prazosin and the α_2_-adrenergic antagonist yohimbine were used to evaluate the role of α-adrenergic receptors in the CHA-induced contraction.

**Fig. 5:**
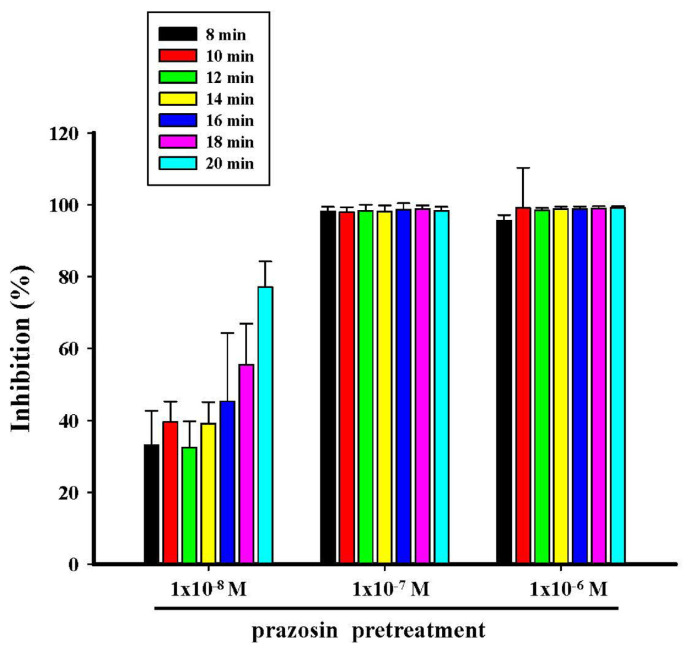
Effects of different concentrations of prazosin pretreatment on CHA-induced contraction of the isolated the rat epididymal vas deferens. A represented the contraction trace of isolated rat epididymal vas deferens and the contraction force (g) change of prazosin (1 × 10^−8^ M ~1 × 10^−6^ M) pretreatment on CHA (1 × 10^−4^ M)-induced contraction (5B). Prazosin pretreatment entirely inhibited the CHA-induced contraction.

**Fig. 6:**
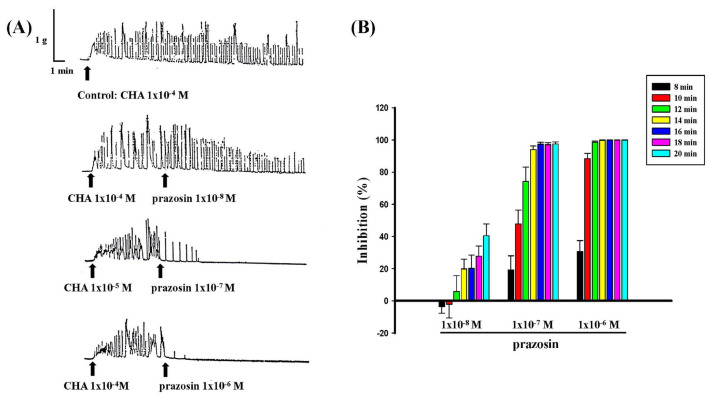
Effects of different concentrations of prazosin on CHA-induced contraction of the isolated rat epididymal vas deferens. 6A represented the contraction trace of isolated rat epididymal vas deferens and the contraction force (g) change in CHA and prazosin treatment. Prazosin (1 × 10^−8^ M ~1 × 10^−6^ M) treatment inhibited the CHA (1 × 10^−4^ M)-induced contraction in a dose-dependent manner (6B).

Different concentrations of prazosin (1 × 10^−8^ M−1 × 10^−6^ M) antagonized the contraction produced by CHA (1 × 10^−4^ M) both in pretreatment and treatment, as shown in Figs. 5 and 6. Prazosin (1 × 10^−7^ M and 1 × 10^−6^ M) pretreatment almost entirely inhibited CHA (1 × 10^−4^ M)-induced contraction (Fig. 5). Prazosin (1 × 10^−7^ M or 1 × 10^−6^ M) was administered afterward, the contraction of CHA (1 × 10^−4^ M) was almost completely inhibited at the end of the fourteen and 12 min, respectively (Fig. 6).

**Fig. 7:**
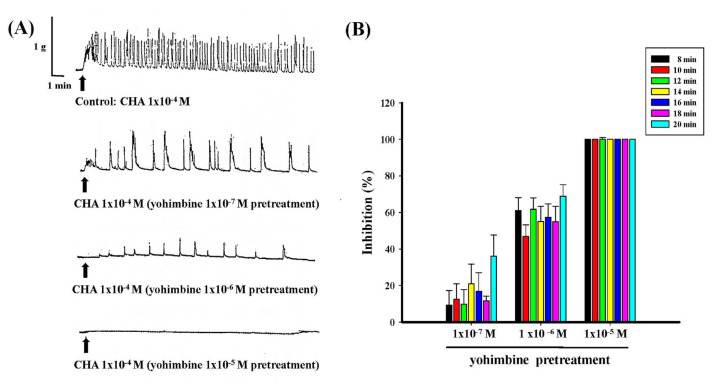
Effects of different concentrations of yohimbine pretreatment on CHA-induced contraction of the isolated rat epididymal vas deferens. 7A represented the contraction trace of isolated rat epididymal vas deferens and the contraction force (g) change in CHA (1 × 10^−4^ M) and yohimbine 1 × 10^−7^ M ~1 × 10^−5^ M) pretreatment. Yohimbine pretreatment dose-dependently inhibited the CHA-induced contraction (7B).

**Fig. 8:**
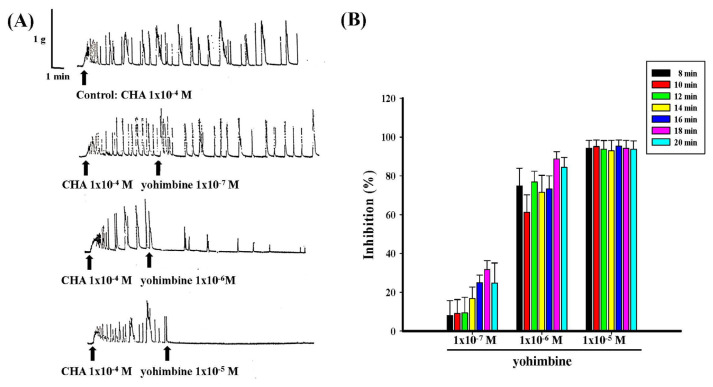
Effects of different concentrations of yohimbine treatment on CHA-induced contraction of the isolated rat epididymal vas deferens. 8A represented the contraction trace of isolated rat epididymal vas deferens and the contraction force (g) change in CHA (1 × 10^−4^ M) and yohimbine (1 × 10^−7^ M ~1 × 10^−5^ M) treatment. Yohimbine treatment inhibited the CHA-induced contraction dose-dependently (8B).

Different concentrations of yohimbine (1 × 10^−7^ M−1 × 10^−5^ M) dose-dependently antagonized the contraction produced by CHA (1 × 10^−4^ M) both in pretreatment and treatment, as shown in Figs. 7 and 8. Yohimbine (1 × 10^−5^ M) pretreatment completely inhibited CHA-induced contraction (Fig. 7), and in yohimbine treatment (1 × 10^−5^ M), the inhibition of CHA-induced contraction could reach more than 90 % (Fig. 8B).

**Fig. 9:**
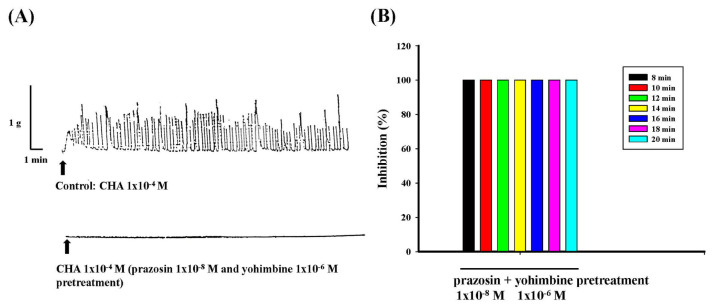
Effects of different concentrations of prazosin and yohimbine pretreatment on CHA-induced contraction of the isolated rat epididymal vas deferens. Prazosin (1 × 10^−8^ M) and yohimbine (1 × 10^−6^ M) pretreatment completely inhibited CHA (1 × 10^−4^ M)-induced contraction.

Prazosin (1 × 10^−8^ M) and yohimbine (1 × 10^−6^ M) were administered 10 min before CHA (1 × 10^−4^ M); the contractile effect of CHA was entirely antagonized by the combination of prazosin and yohimbine pretreatment, as shown in Fig. 9. Fig. 9A represented the contraction trace and Fig. 9B represented the percentage of inhibition were inhibited by prazosin and yohimbine pretreatment.

### 3.4. Effect of a_1A_-adrenergic antagonist WB4101 or a_1B_-adrenergic antagonist CEC pretreatment on the CHA-induced contraction of the isolated rat proximal epididymal rat vas deferens

**Fig. 10:**
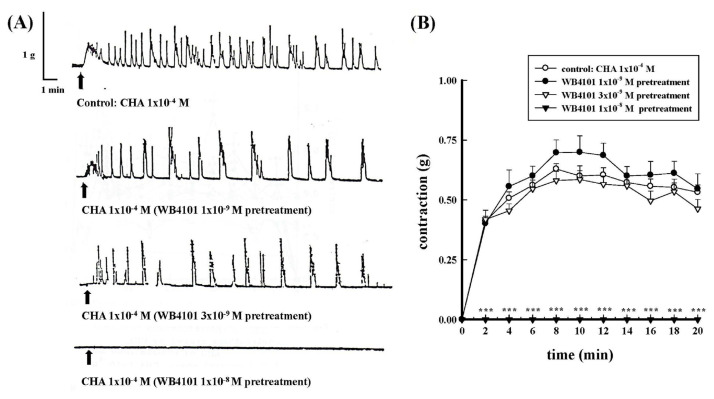
Effects of different concentrations of WB4101 pretreatment on CHA-induced contraction of the isolated rat epididymal vas deferens. 10A represented the contraction trace of isolated rat epididymal vas deferens and the contraction force (g) change in CHA (1 × 10^−4^ M) and WB4101 pretreatment (10B). 1 × 10^−8^ M WB4101 completely inhibited CHA-induced contraction, ***P < 0.001 compared with the CHA group.

WB4101 is a selective α_1A_-adrenergic receptor antagonist. As shown in Fig. 10, 1 × 10^−8^ M WB4101 completely inhibited the 1 × 10^−4^ M CHA-induced contraction; however, the lower concentrations (1 × 10^−9^ M and 3 × 10^−9^ M) of WB4101 did not show significant inhibition of the CHA-induced contraction.

**Fig. 11:**
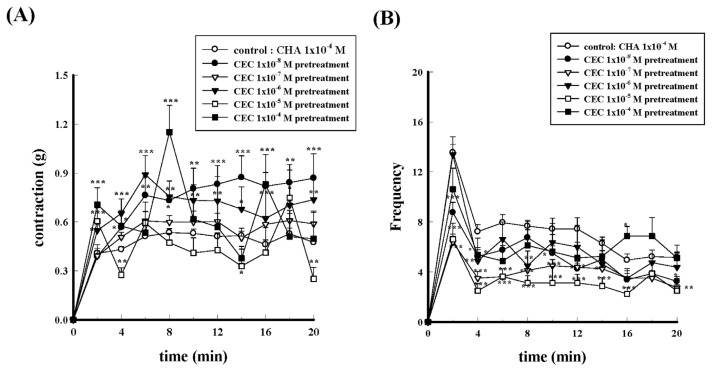
Effects of different concentrations of CEC pretreatment on CHA-induced contraction of the isolated rat epididymal vas deferens. Different concentrations of CEC (1 × 10^−8^ M~1 × 10^−4^ M) pretreatment inhibited CHA (1 × 10^−4^ M)-induced contraction (11A) and frequency (11B) in a dose-dependent manner. *P < 0.05, **P < 0.01 and ***P < 0.001 compared with the CHA group.

CEC is an irreversible α_1B_-adrenergic receptor antagonist. Different concentrations (1 × 10^−8^ M−1 × 10^−4^ M) of CEC pretreatment did not show significant inhibitory effects on CHA-induced contraction in contraction force (g) change, as shown in Fig. 11A. However, different concentrations of CEC (1 × 10^−8^ M−1 × 10^−4^ M) pretreatment inhibited the contraction frequency of isolated vas deferens produced by CHA, as shown in Fig. 11B.

### 3.5. Effects of reserpine on the CHA-induced contraction of the isolated rat proximal epididymal rat vas deferens

**Fig. 12:**
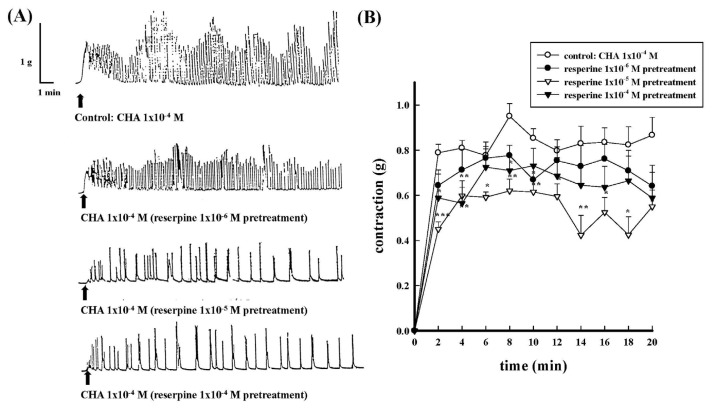
Effects of different concentrations of reserpine pretreatment on CHA-induced contraction of the isolated rat epididymal vas deferens. 12A represented the contraction force (g) change in isolated rat epididymal vas deferens and the contraction frequency change in reserpine (1 × 10^−6^ M ~1 × 10^−4^ M) pretreatment (12B). With increasing concentration of reserpine pretreatment showed a dose-dependent inhibition on CHA (1 × 10^−4^ M)-induced contraction. *P < 0.05, **P < 0.01 and ***P < 0.001 compared with the CHA group.

Reserpine depletes neurotransmitters such as norepinephrine, dopamine, and serotonin in nerve terminals by inhibiting the vesicular monoamine transporter. Different concentrations of reserpine (1 × 10^−6^ M−1 × 10^−4^ M) were administered 2 h before CHA (1 × 10^−4^ M) was administered. Fig. 12A represented the contraction trace of CHA-induced contraction under reserpine pretreatment. Different concentrations of reserpine (1 × 10^−6^ M−1 × 10^−4^ M) pretreatment dose-dependently attenuated CHA-induced contraction in the first 4 min, as shown in Fig. 12B.

## 4. Discussion

While around 30 times sweeter than sucrose, cyclamate has a superior taste profile when combined with saccharin in a 10:1 mixture used in food and drinks. Long-term intake of the saccharin and cyclamate mixture affects biochemical parameters related to metabolic function and appears to increase oxidative stress in healthy and type 2 diabetic patients in a dose-dependent manner [16]. Previous reports indicated CHA could induce atrophy of rat testes and affect rat fertility [12–14], as well as contract rat vas deferens [15]. To date, the mechanism of CHA-induced vas deferens contraction remains poorly understood due to the limited number of studies. In vivo experiments in cats, CHA was first found to be an indirectly acting sympathomimetic agent [17]. In rats, it was also found that CHA induced a hypertensive effect, mainly due to the direct activation of sympathetic receptors [18] or indirectly through the release of catecholamine. In addition, during continuous CHA infusion, tachyphylaxis occurs, which may be attributed to the rapid reuptake of released norepinephrine by sympathetic neurons at low doses and to the depletion of stored norepinephrine at high doses [18].

According to literature [19–22], norepinephrine (NE) produces a dose-dependent systolic enhancement in rat vas deferens, and this systolic reaction is a two-phase systolic reaction, i.e., it consists of a phasic phase and a tonic phase. The present study found that the contractile pattern of CHA on rat epididymal vas deferens was similar to that of NE. The results presented here showed that CHA also caused dose-dependent contractions of the rat epididymal portion of the vas deferens. However, the highest CHA concentration (1 × 10^−1^ M) could quickly produce a strong phasic contraction (0–2 min) and rapidly return to baseline without a twitch response as shown in Fig. 1A. Why the tonic phase completely disappeared at the highest concentration of CHA was a curious question. Testicular effects of CHA might be due to the direct action on the seminiferous epithelium [23]. Furthermore, in the long-term toxicity test, CHA HCl (2000 or 6000 PPM, orally administered) led to testicular changes, including the form of atrophy (bilateral degeneration), minimal spermatogonial content of the tubules; and even a decrease in the mobility of the spermathecae [24,25]. Moreover, previous report indicated that CHA acted directly on the testis and that its primary cellular target was the Sertoli cell [24].

Moreover, there was evidence for a functional α_1A_- (α_1C_-) adrenoceptor-mediated contraction of the rat epididymal vas deferens [26]. In our present study, non-selective α_1_-adrenergic antagonist prazosin significantly antagonized CHA-induced contractions, indicating that α_1_-adrenoceptor mediated the contractile response to CHA in rat epididymal vas deferens. This result was consistent with the report that the α-adrenergic antagonists phentolamine and phenoxybenzamine blocked CHA-induced contraction [15]. In addition, the hypertensive effect of CHA was mediated through the activation of α-adrenergic receptors [28]. It had been reported that CHA elicited higher blood pressure and increased cardiac contractile force by releasing endogenous catecholamine [27]. A similar result could be obtained from our studies, which showed that the attenuation of the CHA-evoked contraction was observed after preincubation with reserpine for 2 h.

Furthermore, from our present in vitro studies, treatment or pretreatment with yohimbine, a presynaptic α_2_-antagonist, or the combination of yohimbine and prazosin markedly antagonized CHA-induced contractions, indicating that CHA might directly act on postsynaptic α_1_-adrenoceptors and affect presynaptic α_2_-adrenoceptors to release NE.

Previous reports indicated that rat vas deferens contained approximately equal numbers of α_1A_- and α_1B_-adrenergic receptor binding sites [28]. Thus, from our present studies, both the α_1A_-adrenergic antagonist WB4101 and the α_1B_-adrenergic antagonist CEC [28] reduced either frequencies or tension responses of CHA, indicating that the effect of CHA was dependent on both α_1A_- and α_1B_-adrenergic receptor, but α_1A_-played more important role than α_1B_-adrenergic receptor because WB4101 could block the responses of CHA in low concentration.

Voltage-dependent L-type Ca^2+^ channels (VDCCs) appeared to mediate CHA–induced contractions because our data showed that incubation with a Ca^2+^-free solution and the VDCC blocker, nifedipine, attenuated the response of CHA. Many research reports pointed out that stimulation of α_1_- adrenoceptors was able to mediate contraction of smooth muscle by raising intracellular calcium [29,30], and α_1A_-adrenoceptors might control voltage-sensitive channels directly through a guanine nucleotide regulatory protein or indirectly through effects on ionic permeability, allowing depolarization and channel opening [31]. Furthermore, it has been well-established that Ca^2+^ triggers the transmitter to be released from synaptic knobs, and a transmitter elicits the calciuminflux in smooth muscle [32]. Thus, previous data revealed that CHA acted on both postsynaptic α_1_- and presynaptic α_2_-adrenoceptors), so we inferred that the calcium source might have a relation to these two parts.

## 5. Conclusions

**Fig. 13:**
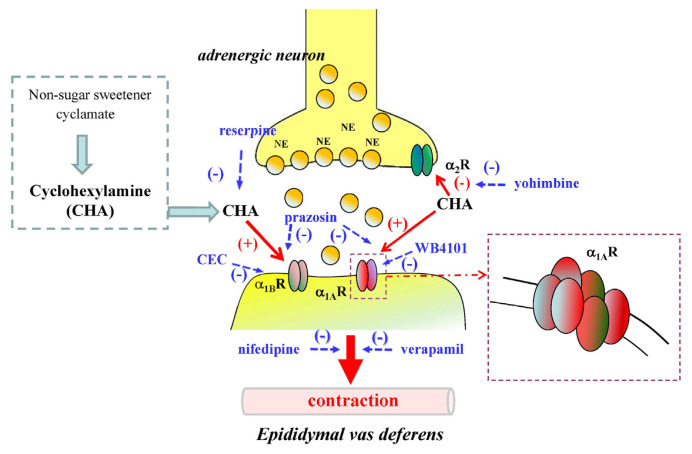
Proposed action mechanism of cyclohexylamine (CHA)-induced contraction of rat epididymal vas deferens. CHA-induced contraction on the isolated rat epididymal vas deferens may be partially via adrenergic α_1A_- α_1B_ and α_2_-receptors that are calcium-dependent. α_1A_R: α_1A_-adrenoceptor; α_1B_R: α_1B_-adrenoceptor; α_2_R: α_2_-adrenoceptor; CEC: chloroethyl clonidine; CHA: cyclohexylamine; NE: norepinephrine; (+): stimulation; (−): inhibition.

CHA directly acts on postsynaptic α1-adrenoceptor, particularly the α_1A_-subtype and presynaptic α_2_- adrenoceptor to release endogenous catecholamines. These effects of CHA on the contraction of rat proximal epididymal vas deferens are calcium-dependent. The proposed action mechanism of cyclohexylamine (CHA)-induced contraction on epididymal vas deferens of rat shows in Fig. 13.
